# Hybrid Adaptive Lossless Image Compression Based on Discrete Wavelet Transform

**DOI:** 10.3390/e22070751

**Published:** 2020-07-09

**Authors:** Roman Starosolski

**Affiliations:** Department of Algorithmics and Software, Silesian University of Technology, 44-100 Gliwice, Poland; rstarosolski@polsl.pl

**Keywords:** lossless image compression, predictive coding, transform coding, hybrid transform, entropy estimation, discrete wavelet transform, reversible denoising and lifting step, step skipping, screen content coding, JPEG 2000

## Abstract

A new hybrid transform for lossless image compression exploiting a discrete wavelet transform (DWT) and prediction is the main new contribution of this paper. Simple prediction is generally considered ineffective in conjunction with DWT but we applied it to subbands of DWT modified using reversible denoising and lifting steps (RDLSs) with step skipping. The new transform was constructed in an image-adaptive way using heuristics and entropy estimation. For a large and diverse test set consisting of 499 photographic and 247 non-photographic (screen content) images, we found that RDLS with step skipping allowed effectively combining DWT with prediction. Using prediction, we nearly doubled the JPEG 2000 compression ratio improvements that could be obtained using RDLS with step skipping. Because for some images it might be better to apply prediction instead of DWT, we proposed compression schemes with various tradeoffs, which are practical contributions of this study. Compared with unmodified JPEG 2000, one scheme improved the compression ratios of photographic and non-photographic images, on average, by 1.2% and 30.9%, respectively, at the cost of increasing the compression time by 2% and introducing only minimal modifications to JPEG 2000. Greater ratio improvements, exceeding 2% and 32%, respectively, are attainable at a greater cost.

## 1. Introduction

Due to large and ever-growing sizes and quantities of images produced in the present day, compression is crucial for picture archiving and communication systems (PACSs). Lossless image compression is employed for medical images used for diagnostic purposes (in many cases, lossy compression of such images is forbidden by law [1,2]). Furthermore, lossless compression is required when we are unsure whether discarding of information contained in an image is applicable or not, which frequently happens when transmitting images over a network or directly from an acquisition device. Therefore, lossless image compression algorithms are often the only alternative to not using compression at all. Although compression ratios (bitrates) achieved by lossless image compression algorithms are not as good as those achieved by lossy algorithms, they allow for multifold increases in transmission media bandwidths and mass storage capacities of PACSs. For the above reasons, and in response to practical demands, many algorithms have been developed and adopted as international standards. JPEG 2000 is the most widely known such standard for lossy and lossless compression of image and volumetric data [3,4,5]. It is utilized, among others, in medicine and it is included in the DICOM standard [6].

Image compression algorithms exploit either pixel-wise prediction (like in the JPEG-LS [7,8]) or two-dimensional transforms of image regions that may be as small as 2 × 2 pixels (in the case of JPEG XR [9,10]) or as big as the entire image (e.g., in JPEG 2000). JPEG 2000 is transform based. Its core component is a discrete wavelet transform (DWT) [11,12], constructed using lifting steps (LSs) [13,14], that decomposes an image into subbands, which are then independently entropy coded. It is believed that the use of prediction that is not supported by additional knowledge does not improve substantially the lossless compression ratios of DWT subbands [15]. Our experiments also confirm that a simple applying of prediction to DWT subbands does not result in substantial bitrate improvements. On the other hand, as a result of previous research we have obtained premises that DWT can be effectively combined with prediction by the use of additional techniques, namely, the reversible denoising and lifting step (RDLS) and step skipping [16,17,18].

The main contribution of this paper consists in proposing a new hybrid transform for lossless image compression that exploits DWT and prediction. In the new transform, prediction is applied to subbands obtained with DWT modified using RDLS with step skipping and, to construct it in an image-adaptive way, heuristics and entropy estimation are employed. We evaluated the transform effects on compression ratios of lossless JPEG 2000 for a large and diverse set of test images. We found that RDLS with step skipping allowed effectively combining DWT with prediction. Using the hybrid transform, we nearly doubled the compression ratio improvements that could be obtained without prediction. Especially good results were obtained for non-photographic (screen content) images. Considering that in some cases it might be better to apply prediction instead of DWT (modified using RDLS with step skipping or unmodified), we proposed a couple of practical compression schemes with various tradeoffs, which are practical contributions of this study.

The remainder of this paper is organized as follows. In the next section, after a note on predictive and transform coding (Section 2.1), we briefly describe the reversible DWT employed by JPEG 2000 (Section 2.2) and DWT modified using RDLS with step skipping (Section 2.3). The main new contributions of this paper are in Section 2.4, Section 2.5 and Section 3.3. The hybrid transform that combines DWT with prediction is proposed in Section 2.4 and two heuristics for image-adaptive constructing of the new transform are presented in Section 2.5. The experimental procedure and test image set are described in Section 2.6. Experimental results are reported and discussed in Section 3, where we first investigate the effects of integrating DWT with prediction (Section 3.1). Next, because we are most interested in the effects worthwhile from a practical standpoint, we employed entropy estimation of compression ratio to reduce the compression time (Section 3.2). Based on observations from Section 3.1 and Section 3.2, we propose and evaluate new and efficient compression schemes in Section 3.3. Section 4 summarizes the findings. The list of all symbols used in the equations can be found in the Nomenclature section before the References section. 

## 2. Materials and Methods 

### 2.1. Predictive and Transform Coding

Efficient image compression employs one of the following approaches: Predictive coding or transform coding. In a predictive algorithm, for each pixel, we use a function (called a predictor) to predict the intensity of the pixel. Image pixels are processed in a certain order, e.g., in the raster-scan order. The predictor is usually a simple linear or non-linear function that uses only a small number of already processed nearest neighbors of the pixel to predict its intensity. We then calculate prediction errors, i.e., differences between actual and predicted pixel intensities, and encode the prediction errors instead of encoding pixels. In a typical case, the entropy of prediction errors is significantly smaller than the entropy of image pixels. Fast and simple image compression algorithms, like JPEG-LS, are predictive. JPEG-LS uses a simple fixed non-linear predictor (named MED, see Table 1 in Section 2.4). There also are algorithms that select a predictor for an image or its fragment from a set of candidate predictors or that adjust parameters of generic predictors for adaptively determined image fragments. The latter approach is used in the state-of-the-art MRP algorithm [19]. However, its time complexity may be too high for most practical applications. Predictive algorithms are used primarily for lossless compression.

Another method of making the image data more easily compressible, the transform or transform-based coding, applies a two-dimensional transform to the entire image or to an image split into fragments. The entropy of transformed pixels usually is much smaller than the entropy of the original image. An additional advantage of transform coding is that essentially the same algorithm can perform both lossy and lossless compression. The two most popular transforms in image compression are discrete cosine transform (DCT) and DWT. The former is employed by the classic lossy JPEG algorithm, which is still the most popular algorithm for lossy image compression [20], and by the more recent JPEG-XR that is capable of lossy and lossless coding. DWT is employed by JPEG 2000 that also has lossy and lossless variant; the DWT variant for lossless compression is presented in the next section.

Generally, practical algorithms for compression of images do not combine predictive and transform coding. However, in MPEG family standards [21] a hybrid approach, which combines transform coding with a form of prediction, is successfully used to compress video frame sequences. Compared to the typical predictive coding of static images, prediction in MPEG is performed differently and is much more complex. For fragments of the frame (macroblocks), we find in neighboring frames, at some distance from the macroblock subjected to prediction, the most similar fragment (or several such fragments). Starting from the MPEG4-AVC standard [22], also the current frame may be used for prediction. In such a case, MPEG4-AVC allows exploiting only neighboring samples in a single line or column, whereas the upcoming VVC standard [23] defines several modes of using macroblocks from the current frame for prediction. Then the transform coding is applied to the macroblock prediction error, but the so-called motion vectors describing the locations of the found fragments used for prediction must also be encoded (in VVC, before prediction we may additionally apply affine transforms to the found fragments).

### 2.2. Reversible Discrete Wavelet Transform

DWT employed in image compression algorithms decomposes an image into subbands that are easier to encode because their characteristics are well-defined and narrow. In lossless JPEG 2000, each subband is compressed independently of the others [3,4]. For brevity we characterize here only certain aspects of the lifting-based reversible DWT with the Cohen Daubechies Faveau (5, 3) wavelet filter, which is used in lossless JPEG 2000 compression. For further details of this DWT variant the reader is referred to [16,17]. The description of other DWT variants, details of application of DWT in JPEG 2000, and the JPEG 2000 standard may be found in [3,4,11,15].

The one-dimensional DWT (1D-DWT) transforms in-place a discrete signal *S* = *s*_0_ *s*_1_ *s*_2_ … *s_q_*_−1_ of finite length *q* into a low-pass filtered subband *L* that represents the original signal’s low-frequency features and a high-pass filtered subband *H* (containing high-frequency features) that, along with *L*, allows perfect reconstruction of the original signal. *S* is transformed in three steps. First, in the prediction step, we perform the high-pass filtering of odd samples (hereafter, the parity of a sample or pixel is determined by its location and not its value) by applying in place the below LS to each of them.

(1) $$s_{x}\leftarrow s_{x}-\left\lfloor (s_{x-1}+s_{x+1})/2\right\rfloor ,$$

where ⌊v⌋ denotes the greatest integer less than or equal to *v*. Note that the name of this LS (the “prediction step”), may be confused with the operation (“prediction”) performed by predictive image compression algorithms to make image data more easily compressible. Another LS is then applied in place to each even sample during the update step:

(2) $$s_{x}\leftarrow s_{x}+\left\lfloor (s_{x-1}+s_{x+1}+2)/4\right\rfloor .$$

Finally, in the reorder step, we reposition even samples to the lower half of the original signal, preserving their ordering (sample $s_{x}$ is moved to $s_{x/2}$), and odd samples are moved to the upper half. We obtain separate subbands, *L* and *H*, respectively. 

**Figure 1:**
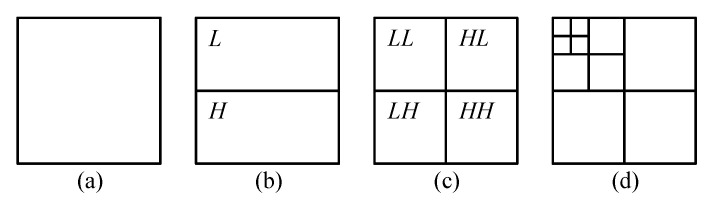
One-level 2D-DWT (**a**–**c**) and three-level 2D-DWT (**d**).

The two-dimensional DWT (2D-DWT) for an image is obtained by first applying 1D-DWT to each image column, which results in *L* and *H* subbands of the image. Then by applying 1D-DWT to rows, we obtain the one-level DWT, consisting of *LL*, *HL*, *LH*, and *HH* subbands; see Figure 1a–c. We will call a subband belonging to a subband pair (*L*, *H*), (*LL*, *HL*), or (*LH*, *HH*) complementary to another subband from the same pair.

The higher-level DWT is obtained by Mallat decomposition [12]. The *l*+1-level transform is obtained by applying the one-level transform to the *LL* subband of *l*-level transform (Figure 1d). Note, that not all subbands that are created during the DWT execution remain after its completion. *L*, *H*, and at all transform levels except the highest *LL* are further transformed in place. We will call them the temporary subbands, whereas others will be called the final subbands.

### 2.3. Application of Reversible Denoising and Lifting Steps with Step Skipping to DWT

**Figure 2:**
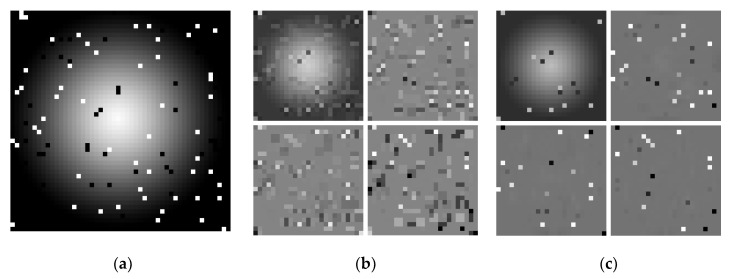
A noise-contaminated image (**a**) and its one-level DWT (**b**) and RDLS-DWT (**c**).

An unwanted side effect of LS is that the sample being modified by LS (filtered by LS in the case of DWT) gets contaminated by noise from other samples. Hence, noise gets propagated between DWT subbands. Figure 2 illustrates this effect. Figure 2a shows a noise-contaminated image in which 5% of pixels are replaced with black or white pixels. Nearly every noisy pixel in the image results in an artifact in each DWT-transformed subband (Figure 2b). Because JPEG 2000 encodes the DWT subbands independently, the noise propagation increases the amount of information that has to be encoded and, thus, worsens the compression ratios.

To limit the noise propagation, in [16] we proposed RDLS, which is built based on LS, and applied it to DWT obtaining RDLS-DWT. RDLS prevents the noise propagation by exploiting denoising filters while retaining other desirable effects and properties of LS (and consequently of the lifting-based transform). In brief, we replaced the prediction (Equation (1)) and update (Equation (2)) LSs with their RDLS-modified counterparts (Equations (3) and (4), respectively):

(3) $$s_{x}\leftarrow s_{x}-\left\lfloor (s_{x-1}^{d}+s_{x+1}^{d})/2\right\rfloor ,$$

(4) $$s_{x}\leftarrow s_{x}+\left\lfloor (s_{x-1}^{d}+s_{x+1}^{d}+2)/4\right\rfloor .$$

where $s_{i}^{d}$ is the denoised sample $s_{i}$ obtained using a deterministic denoising filter. Denoising is not an in-place operation, i.e., computing $s_{i}^{d}$ does not alter $s_{i}$. For the denoising of a sample of a specific parity, we used samples of the same parity only. The same denoising filter was used while filtering all samples that, after the reorder step, are placed in a specific subband at a specific transform level. So only one filter number per subband needed to be transmitted to the decoder. Filters were selected in an image-adaptive way. (Filter selection methods for the hybrid variant of the transform are discussed in Section 2.5). The example effect of RDLS-DWT is presented in Figure 2c, where the median filter with a 3 × 3 pixel window was used for all subbands. We can see that noisy pixels remain in the subband. They are placed in by the reorder step rather than getting propagated to other subbands or causing artifacts.

RDLS has very interesting properties (discussed in [16,17]). Despite the inherently lossy nature of denoising, RDLS-DWT is perfectly and easily invertible. The inverse prediction and update steps of RDLS-DWT are presented in below Equations (5) and (6), respectively:

(5) $$s_{x}\leftarrow s_{x}+\left\lfloor (s_{x-1}^{d}+s_{x+1}^{d})/2\right\rfloor ,$$

(6) $$s_{x}\leftarrow s_{x}-\left\lfloor (s_{x-1}^{d}+s_{x+1}^{d}+2)/4\right\rfloor .$$

The RDLS-modified transform is more general than the original one. As its special case, we may obtain the unmodified transform by using a special denoising filter, denoted as None, for which $s_{i}^{d}=s_{i}$ [16]. Another special filter, the Null filter, for which $s_{i}^{d}=0$, was proposed in [18] for the RDLS-modified color space transforms. It was found effective for several reversible color space transforms (for classic RCT [4] and YCoCg-R [24] as well as for two simplified ones introduced in [25,26]). The Null filter allows for practically skipping the transform step, thus providing an additional mechanism of adaptation of the RDLS-modified transform when overall transform effects (both the unwanted side effects and normally expected ones) result in worsening of the compression ratio. Thanks to Null, RDLS may also be effective for noise-free data, i.e., beyond its originally intended area of applicability.

In [16] we found that the noise filtering in RDLS-DWT significantly improved the lossless JPEG 2000 bitrates of non-photographic images (by about 14% on average). It was the most effective when applied to some subbands only. Since, in some cases, the best bitrates were obtained when the DWT stage of JPEG 2000 was skipped, we suspected that the optimum might be in between skipping and applying DWT. The prediction and update steps in RDLS-DWT may be skipped using the Null filter, which turns them into $s_{x}\leftarrow s_{x}$. However, the non-lifting reorder step limited the freedom to skip selected parts of the transform. Therefore, in [17] we proposed the skipped-steps DWT (SS-DWT) and skipped-steps RDLS-DWT (RDLS-SS-DWT) obtained from DWT and RDLS-DWT, respectively, by allowing to skip any step of the transform computation including the reorder step. We found that the reorder step should be skipped if and only if Null was used for both complementary subbands. Compared to RDLS-DWT, SS-DWT allowed obtaining similar improvements at a smaller cost, whereas RDLS-SS-DWT resulted in increased bitrate improvements (up to about 17.5%).

Interestingly, extensions of the second part of the JPEG 2000 standard [27] allow obtaining, in conformance with the standard, certain fixed variants of SS-DWT (FIX1 and FIX2 proposed in [17]). The compression scheme presented in [28] results in an image decomposition similar to the one produced by FIX2. On the other hand, SS-DWT is, in some respects, similar to nonlinear wavelet transforms [29,30]. However, these transforms could not result in skipping of the update or reorder steps, nor were they effectively resulting in skipping of the prediction step.

So far, besides the Null and None special filters, experiments with RDLS were performed using simple linear and nonlinear denoising filters. The latter [16] were found to be effective for lossless JPEG 2000. Based on those findings, in this work we exploited two median filters (with 5 × 5 and 3 × 3 pixel windows) and two milder filters with 3 × 3 pixel windows (denoted RCRS-1 and RCRS-2 in [16]).

### 2.4. Hybrid Transform that Combines DWT with Prediction

It is believed that the use of prediction that is not supported by additional knowledge (like knowledge on the symmetry in medical images) does not improve significantly the lossless compression ratios of DWT subbands—which is confirmed by, e.g., Bruylants et al. [15]. Yet the characteristic of RDLS-SS-DWT subbands is different than in the case of DWT subbands. If a harsh filter is used in RDLS, and especially if transform steps are skipped, then it may be closer to the characteristic of an untransformed image than to the characteristic of subbands of unmodified DWT. As a consequence, prediction may improve the compression effects of RDLS-SS-DWT subbands.

To test it, we proposed a new hybrid transform RDLS-SS-DWT+Pred that combines DWT with prediction. In RDLS-SS-DWT+Pred, to each final subband of RDLS-SS-DWT-transformed image a predictor may be applied, which is selected from a set of available predictors. The same predictor is applied to all samples of the subband and we allow to not use prediction if it is not beneficial for the subband; different predictors may be used for different subbands. Constructing this transform efficiently in an image-adaptive way is not straightforward. The naive method of constructing RDLS-SS-DWT+Pred could consist of two stages: First, in an image-adaptive way, the RDLS-SS-DWT would be constructed (i.e., by selecting RDLS filters for a given image, like in [17]) and then, for each of its final subbands, the predictor could be selected that gives the greatest improvement in the compression ratio of this subband (or prediction could be skipped if it does not improve the subband compression effects). The naive method may obviously be able to improve the compression ratios of some subbands, but it will not allow exploiting of the full potential of the hybrid transform. Filters that are optimal for RDLS-SS-DWT may not be optimal when we apply prediction to final subbands. The selection of filters and predictors is interdependent; hence, RDLS filters for RDLS-SS-DWT+Pred should be selected taking into account the subsequent prediction of final subbands affected by the filter selection. On the other hand, the aim of the research was to find a method of compression useful from a practical point of view, so it cannot be too expensive. There are more subbands for which the RDLS filter needs to be selected (all subbands including temporary ones) than the subbands that require selecting a predictor (only final subbands). In most cases, however, the RDLS filter selected for one subband affects the selecting of predictors of multiple final subbands. During the image-adaptive construction of the transform, we need to check the effects of using the predictor for the subband much more often than the effects of filter selection. For that reason, we used a fast estimation of the subband compression effects to select the predictor, and this selection method was taken into account in the method of RDLS filter selection (see the next section for heuristics of the selection of RDLS filters). In RDLS-SS-DWT+Pred, to each final subband we applied the predictor selected from a set of candidate predictors, which resulted in the smallest memoryless entropy of prediction errors. We calculated the memoryless entropy using Equation (7):

(7) $$-M\sum_{i=\mathrm{MinPE}}^{\mathrm{MaxPE}}p_{i}\log_{2}p_{i},$$

where *M* is the number of samples in the subband, MinPE and MaxPE are the smallest and the greatest prediction errors obtained, respectively, *p_i_* is the probability of occurrence of the prediction error value *i* in the subband, and we assume that $0\log_{2}0=0$. The memoryless entropy proved to be a sufficient estimator of the effects of subband compression from a practical point of view in [17], where it was used to select RDLS filters and obtained slightly worse effects than the actual context entropy coder of the actual compression algorithm (JPEG 2000) while being two orders of magnitude faster.

The set of candidate predictors exploited in this research is presented in Table 1, in which by W we denote the left-hand neighbor of the pixel being predicted; N, upper neighbor; and NW, upper-left one. The NOP predictor allows skipping prediction if all the actual predictors fail to improve the bitrate of the subband estimated using the memoryless entropy. Other predictors could not be computed for some subband edges; in such a case we used the NOP predictor for the top left corner of the subband, LEFT for the top row, and UPPER for leftmost subband column. The selected predictors should be transmitted to the decoder as side information along with the compressed image. The size of this data is negligible (three bits per final subband if we allow all predictors from Table 1 or one bit if we allow only NOP and MED).

**Table 1:** Predictors used in the research.

Predictor	Prediction
NOP	0
MED	median(W, N, W + N − NW)
AVG	(W + N)/2
LEFT	W
UPPER	N

### 2.5. Filter Selection Heuristics for the Hybrid Transform

To construct RDLS-SS-DWT+Pred for an image we must select denoising filters—one for each subband including temporary ones, i.e., six filters for each transform level. Naturally, the filter selection should be made taking into account the way predictors are chosen for final subbands. Even for low transform levels, performing a full search would rule out the practical application of this transform. Therefore, for the image-adaptive selection of denoising filters, we used two variants of a heuristic, which were based on heuristics used in [17]. The new heuristic (NH) imposed fewer constraints on the resulting transform and was primarily used to evaluate the effects of combining RDLS-SS-DWT with prediction. It consisted of the two greedy steps A and B presented below:

- For each of the denoising filters, check the bitrate obtained for an image, using this filter in RDLS-SS-DWT+Pred steps for all subbands at all transform levels. Then for all subbands at all levels, select the filter that resulted in the best overall bitrate.
- For each transform level *a* (starting from level 1) and for each subband *b* (at a specific level analyzed in the *H*, *L*, *HL*, *HH*, *LL*, and *LH* order), try to find a better filter by checking for each filter (except for the one already selected) the bitrate obtained using this filter for subband *b* at level *a*, while the filters selected so far are used for other subbands. If the Null filter gets selected for a prediction step, then select it also for the complementary update step.

Step A was performed once and then we performed zero or more iterations of step B (the number of iterations is the heuristic’s parameter). Note, that this heuristic does not explicitly decide whether to skip the reorder steps because in RDLS-SS-DWT+Pred they are skipped, if for both complementary subbands the Null filter is selected. Selecting the Null filter for a prediction step implies selecting it for the complementary update step as well. However, the filter for the update step may be later changed again because the order of analyzing subbands makes the heuristic first find a filter for a prediction step and next for its complementary update. The predictors for final subbands are also not explicitly selected by NH (in RDLS-SS-DWT+Pred they are selected for each such subband using Equation (7)). Therefore, after a changing a filter for a given subband by the step B of the heuristic (even changing temporarily when searching for the best filter), the predictors for all the final subbands affected by this change must be selected again.

The time complexity of compression of a *P*-pixel image exploiting NH depends on: *n*, the number of iterations of the step B of the heuristic; *l*, the transform level; *f*, the number of filters; and *p*, the number of predictors. Calculating it we took into account that, while performing the heuristic, changing the filter for a specific subband may require computing of only some other subbands and, for those of them that are final, selecting a predictor and testing the bitrate. The number of the prediction errors that for *f* > 1 are encoded by a single iteration of step B of NH is

(8) $$P\left(4f-\frac{8}{3}\right)\left(1-4^{-l}\right),$$

whereas the number of RDLSs that need to be performed (excluding the costless RDLSs with the Null filter) is

(9) $$P(\left(\frac{86}{9}-\frac{1}{3}2^{5-2l}l-\frac{43}{9}2^{1-2l}\right)(f-2)+\frac{104}{9}-\frac{1}{3}2^{5-2l}l-\frac{13}{9}2^{3-2l}).$$

The above components of the heuristic’s complexity (Equations (8) and (9)) for increasing *l* quickly converge to maxima and other complexity components are proportional to them. We will estimate the upper complexity bound assuming the infinite *l*.

The total cost of compression exploiting step A and *n* iterations of step B of NH is smaller than the sum of: $T_{\mathrm{fs}}^{\mathrm{NH}}$, the cost associated with applying prediction and entropy coding of final subbands created during the operation of the heuristic; $T_{\mathrm{as}}^{\mathrm{NH}}$, the cost of applying RDLSs while computing all subbands including temporary ones; and the cost of remaining operations that must be done by the compressor (like the file i/o).

(10) $$T_{\mathrm{fs}}^{\mathrm{NH}}=P\left(f+n\left(4f-\frac{8}{3}\right)\right)\left(c_{\mathrm{enc}}+(p-1)c_{\mathrm{pred}}+pc_{\mathrm{est}}\right),$$

where *p* > 1; *c*_enc_ is the cost of entropy-coding of a single prediction error; *c*_pred_, the cost of predicting a sample; and *c*_est_, the cost of estimating bitrate using Equation (7) per single sample. We took into account that the NOP predictor did not require actual predicting but the bitrate of unmodified samples still must be estimated.

(11) $$T_{\mathrm{as}}^{\mathrm{NH}}=P(\left(\left(\frac{86}{9}(f-2)+\frac{104}{9}\right)n+\frac{8}{3}(f-1)\right)c_{\mathrm{LS}}+\left(\frac{86}{9}n+\frac{8}{3}\right)(f-2)c_{\mathrm{den}}),$$

where *c*_LS_ is the cost of a single LS, and *c*_den_, the cost of denoising of a sample. Since RDLS with the None filter does not involve actual denoising, we expressed the RDLS cost using the separate costs of LS and denoising. Note that the unmodified DWT is obtained in 8*P*/3 LSs (for infinite *l*).

Another heuristic, named the revised heuristic (RH), was proposed in [17] after noticing that the result of step A allows predicting, with a very high probability, which filters would be selected by step B for certain subbands. RH differs from NH in step B only, which is of lower complexity and has two variants applied depending on the filter selected by the step A. Their version for RDLS-SS-DWT+Pred is:

B1. If the Null filter was selected in step A, then for each transform level *a* (starting from level 1) and for subbands *b* at a specific level analyzed in the *H*, *HL*, and *HH* order, try to find a better filter by checking for each filter (except for the one already selected) the bitrate obtained using this filter for subband *b* at level *a*, while the filters selected so far are used for other subbands.
B2. If a filter other than Null was selected in step A, then for each transform level (starting from level 1) and for subbands *b* at a specific level analyzed in the *L*, *HH*, *LL*, and *LH* order, try to find a better filter by checking for each filter (except for the one already selected) the bitrate obtained using this filter for subband *b* at level *a*, while the filters selected so far are used for other subbands. If the Null filter gets selected for *HH*, then select it for the complementary *LH*.

The B2 step in our experiments was never faster than B1, so we estimated the complexity of RH, assuming that B2 was used:

(12) $$T_{fs}^{\mathrm{RH}}=P\left(f+n\left(2f-\frac{2}{3}\right)\right)\left(c_{\mathrm{enc}}+(p-1)c_{\mathrm{pred}}+pc_{\mathrm{est}}\right),$$

(13) $$T_{\mathrm{as}}^{\mathrm{RH}}=P(\left(\left(\frac{40}{9}(f-2)+\frac{58}{9}\right)n+\frac{8}{3}(f-1)\right)c_{\mathrm{LS}}+\left(\frac{40}{9}n+\frac{8}{3}\right)(f-2)c_{\mathrm{den}}).$$

The filters selected by the heuristic should be transmitted to the decoder along with the compressed image. One filter gets selected for each subband (including temporary ones), so the size of this data (three bits per subband if filters are selected from a set of six) is negligible. Interestingly, there are two approaches that might allow avoiding the use of the heuristic and transmitting the selected filters. In the case of images, whose acquisition parameters are known and should be stored along with the compressed image, the detector precision characteristic (DPC) approach [31] may be applied; it constructs the acquisition device model that allows for immediate selection of the denoising filters for the image based directly on the acquisition process parameters. The so-called single image noise level estimation or single image denoising algorithms [32,33] find parameters of a denoising filter for an image based on analysis of this image only and independently of the further processing of this image. This approach might be used instead of a heuristic for any image, but generally it would not be practical considering the tradeoff between the increased decoder complexity and the negligible overhead due to transmitting the filters determined by the coder.

The hybrid transform can be applied instead of DWT in lossless image compression, e.g., wherever the lossless JPEG 2000 is used. Due to exploiting the step skipping, it can lead to different image decompositions into subbands than DWT, which in turn may limit or complicate the use of certain JPEG 2000 features, such as progressive or region of interest coding. On the other hand, in most cases, the image is compressed in order to be then decompressed as an entire image.

### 2.6. Test Data, Experimental Procedure, and Implementations

In experiments, we used the green components of images from a CT2 set [34]. The CT2 is a recent large set of color images that was used in the research on color space transforms based on LSs [25,35] and in our previous research on RDLS-SS-DWT [16,17]. It contains 746 images taken from various sources. Image sizes range from 180 × 117 to 6600 × 5100 pixels. The set is divided into two subsets: Photo, consisting of 499 photographs, and No-photo, consisting of 247 non-photographic images. Non-photographic images are images that are different than the natural continuous-tone images, e.g., they are computer-generated, composed from others (including natural ones), or are screenshots. They may be described as screen-content images and it is worth mentioning that, although photographic images are the most common in real-life PACSs, there currently is a growing interest in compression of screen content images [36].

We used the IRIS-JP3D JPEG 2000 part 10 (JP3D [5]) reference software developed by Tim Bruylants from Vrije Universiteit Brussel (VUB) and Interdisciplinary Institute for BroadBand Technology (IBBT), which is downward compatible with the baseline JPEG 2000 standard [15]. We used IRIS-JP3D as we intended to apply RDLS-SS-DWT+Pred for coding of 2D and 3D data and because in this implementation it was relatively easy to modify the DWT stage. Our implementation of RDLS-SS-DWT+Pred (Supplementary File S1) is available [37] as a patch to IRIS-JP3D. The codec executable utilized in the research was a single-threaded application compiled for x64 target platform using Visual Studio Enterprise, version 15.6.6. Experiments were performed on a computer equipped with an Intel i7-8550U CPU (clock speed 3.96 GHz) and 16 GB RAM. In the experiments, except for replacing DWT with RDLS-SS-DWT+Pred and setting the transform level, we used the default codec settings. The whole image was compressed as a single tile, and we used the three-level decomposition (or 0-level to bypass the DWT stage in the JPEG 2000 coding).

**Figure 3:**
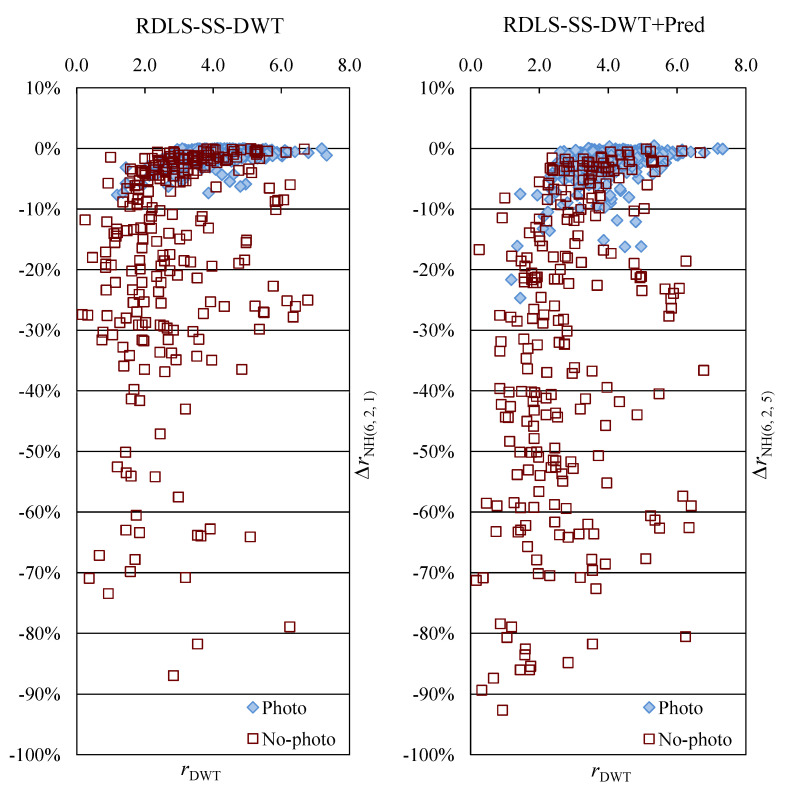
Improvements of bitrates of individual images obtained using RDLS-SS-DWT and RDLS-SS-DWT+Pred plotted against the bitrate of unmodified JPEG 2000.

The bitrate *r*, expressed in bits per pixel (bpp), is calculated using the total size in bytes of the compressed image that includes the compressed file format header and an additional overhead due to transmitting the selected filters and predictors. We introduced modifications to the JPEG 2000 transform stage and then evaluated their effects by analyzing the obtained bitrate changes *∆r* with respect to the bitrate of unmodified JPEG 2000, which was our reference method. The *∆r* is expressed in percentage of the reference method bitrate. In tables, due to the large number of images in the test set, we report averaged bitrates and averaged bitrate changes for Photo and No-photo subsets rather than results for individual images, whereas Figure 3 presents individual images’ results.

NH and RH select RDLS filters by checking their effect on the JPEG 2000 bitrate, which may be achieved either using the actual JPEG 2000 entropy coder or a much faster estimator of the coding effects. We used both approaches. The actual coder was used for initial analysis of the RDLS-SS-DWT+Pred effects, whereas estimation was exploited in practical schemes. As the estimator, we employed the memoryless entropy *H*0 of the image transformed using *l*-level RDLS-SS-DWT+Pred. *H*0 was calculated as a sum of memoryless entropies (Equation (7)) of all RDLS-SS-DWT+Pred subbands that would be independently encoded by JPEG 2000 with an unmodified *l*-level DWT, i.e., 10 subbands for three-level transform, regardless of skipped reorder steps. *H*0 imitates the behavior of the entropy coder of the implementation used, which is unaware of the skipped reorder steps and, thus, may encode a single RDLS-SS-DWT+Pred subband as two or more separate subbands that would be created if the reorder steps were not skipped. *H*0 is used only for image-adaptive construction of RDLS-SS-DWT+Pred; the *r* and *∆r* are in each case calculated from the actual compressed image file sizes.

## 3. Experimental Results and Discussion

### 3.1. Effects of Integrating DWT with Prediction

In Table 2, we present the average bitrate of unmodified JPEG 2000 (*r*_DWT_) and average bitrate changes (*∆r*_variant_) due to applying various variants of DWT. A variant is described by a transform name followed by a method used to obtain the transform parameters. NH(*f*, *n*, *p*) or RH(*f*, *n*, *p*) denote the heuristic employed, where *f* is the number of denoising filters; *n*, number of iterations of the step B of the heuristic (0 means that only step A was performed); and *p*, number of predictors. We used either all six denoising filters described in Section 2.3 or None and Null, the latter resulting in SS-DWT instead of RDLS-SS-DWT. If we used only one predictor, then it was the NOP predictor that effectively disables prediction; otherwise we either employed all five predictors from Table 1 or only the NOP and MED that proved to be the most efficient. By DWT+Pred(5) we denote applying prediction to the subbands of the unmodified DWT (a predictor, for each subband independently, was selected out of all five predictors). RDLS filters were selected by a heuristic based on actual bitrate of the transformed image, but the predictor for each subband was selected based on the memoryless entropy of prediction errors calculated using Equation (7)—both in DWT+Pred(5) and during operation of the heuristic. In the column labeled “Time rel.”, we report the total time of compression (including the heuristic) relative to the time of unmodified JPEG 2000. Thus, this time has no unit of measurement and, for instance, Time rel. of 1.69 (see the last row in Table 2) means that the total compression time of the SS-DWT+Pred, NH(2,0,2) variant was by 69% higher than the time of unmodified JPEG 2000. Time rel. was estimated based on the variant’s time complexity and the actual execution times of elements of JPEG 2000 and the heuristic reported in Table 3; execution times were measured on a computer system used in this research and averaged for several large images from the test set. We report the prediction time for the most complex MED predictor (we assumed that NOP was costless and others were equally complex as MED) and average denoising time per single denoising filter other than the costless None and Null (calculated assuming that all such denoising filters were computed because some operations are common for them).

To assess the effects of the hybrid transform, let us compare the best results obtained with and without prediction, i.e., the ones obtained using two iterations of the heuristic’s step B, all denoising filters, and, in the case of RDLS-SS-DWT+Pred, all predictors (rows 3 and 4 in Table 2). Compared with using RDLS-SS-DWT in JPEG 2000, by exploiting prediction we almost doubled the average compression ratio improvements of both kinds of images. Bitrates of Photo and No-photo images were improved by over 2% and 31%, respectively, which is a very good result, in contrast to applying prediction to the unmodified DWT that did not lead to significant bitrate improvements. Comparing the effects of the above variants for individual images (shown in Figure 3), we see that bitrates of almost all No-photo images were improved noticeably by employing prediction and especially large improvements (e.g., by over 50%) happened much more often than without prediction. When employing prediction, just like without prediction, greater improvements of Photo and No-photo images were observed for those images whose bitrate of unmodified JPEG 2000 was smaller. For instance, using RDLS-SS-DWT+Pred for images whose bitrate of unmodified JPEG 2000 was below 2.2 bpp resulted in bitrate improvement of at least 7.5%, with a single exception of a 5.5% improvement.

The bitrate improvements were very good but came at the expense of a big deterioration in the speed of compression. Fortunately, the extra cost of applying prediction in the hybrid transform is relatively low compared to the extra bitrate improvement due to prediction. We expected to reduce this cost similarly to reducing the cost of RDLS-SS-DWT and SS-DWT in [16,17], i.e., by adjusting the heuristic’s parameters, altering the heuristic itself, and employing entropy estimation for selection of denoising filters. For NH it seemed reasonable to perform step B only once because the improvement of the next iteration was small. Performing only step A (row 6) caused a drop in bitrate improvement of Photo images by a quarter. We may avoid this drop and improve the compression speed by using only one iteration of step B and adjusting other parameters of NH—the number of filters or predictors. It is better to reduce the number of filters because this number has less impact on the bitrate improvement and the cost of using several predictors is much smaller than the cost of using several filters (rows 7–9). The difference in speed of filter and predictor selection is due to employing estimation for selecting predictors and actual coding for filters; also the filters other than Null and None are much slower than predictors (see Table 3). These filters appeared less useful for RDLS-SS-DWT+Pred than they were for RDLS-SS-DWT without prediction. In [17], employing them resulted in increasing bitrate improvements of No-photo images by about 2 percentage points (compared with using only Null and None), whereas here they increased bitrate improvements by below 0.5 percentage points.

The variants discussed so far that use only None and Null filters (rows 8 and 9) seemed the most promising from a practical standpoint. However, their cost is several times greater than the cost of unmodified JPEG 2000 and may be too high for certain practical applications. To decrease the cost, in the next section we investigate an estimation-based filter selection. The compression speed may be also improved by employing RH instead of NH. So far, we evaluated the effects of combining RDLS-SS-DWT with prediction using NH because this heuristic imposes fewer constraints on the resulting transform. However, the negative impact of RH constraints on the bitrate (compare rows 8 and 10) is small as opposed to their positive impact on the compression speed or time complexity, so the practical variant of the hybrid transform should rather be based on RH.

In the last three rows of Table 2 we report results obtained by performing only step A of the heuristic (which is denoted as NH but actually may be NH or RH since they differ only in step B) and using a reduced number of filters or predictors. Naturally, these variants obtained worse improvements for Photo images. Again, the most interesting were variants that used only 2 filters (last 2 rows). Their speed seems acceptable, but employing estimation for filter selection and the heuristic’s step B may result in better overall performance, which is investigated in the next section. Interestingly, because the same filter in the heuristic’s step A was applied to all RDLS-SS-DWT steps, these two variants resulted in either performing unmodified DWT followed by prediction (if the None filter was selected) or effectively skipping DWT (for the Null filter) and applying prediction to untransformed image data. Bitrate improvements of these variants are, especially for No-photo images, close to improvements obtained using one iteration of step B (rows 8 and 9). Considering the poor performance of DWT+Pred(5) (row 2), the question arose as to whether the RDLS-SS-DWT+Pred transform obtained using both steps of the heuristic was indeed a hybrid transform that integrates prediction with DWT or perhaps RDLS-SS-DWT+Pred gets reduced to either applying prediction instead of DWT or using unmodified DWT. Thus, we checked if prediction was actually applied to subbands of RDLS-SS-DWT transformed image data.

**Figure 4:**
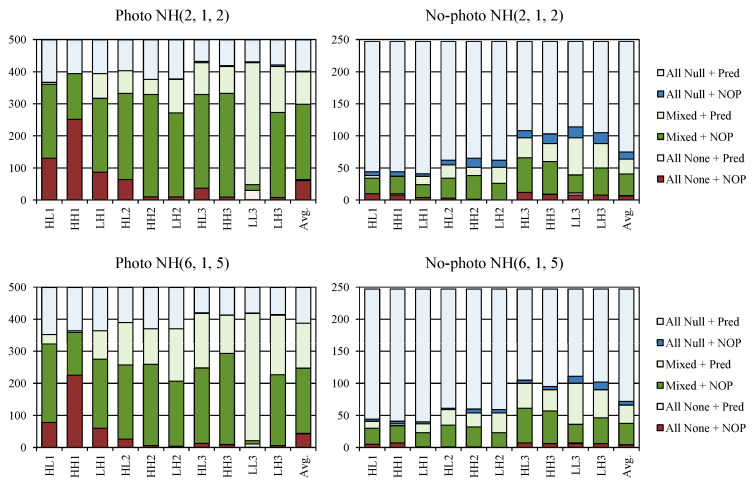
Use of prediction in subbands of various origins.

Figure 4 shows, for each RDLS-SS-DWT subband (denoted by the subband name followed by transform level) and on average for all subbands (denoted Avg.), in how many images the actual prediction was applied depending on the subband origin. We recognized three origins of subbands. By “All None” we denote that all the RDLS-SS-DWT steps used to obtain given subband employed the None filter, so the subband was identical to subband obtained with the unmodified DWT. “All Null” means that the Null filter was used in all steps (and all the reorder steps were skipped); in this case the subband samples were untransformed. “Mixed” means that at least two different filters were used, so the subband was created using RDLS-SS-DWT not reduced to DWT or to transform skipping. Origin name followed by “+ NOP” denotes that the NOP predictor was selected for the subband, so effectively prediction was not applied to the subband. Results for NOP are plotted in a dark color. Otherwise, an actual prediction was applied—which is plotted in a light color and denoted by “+ Pred”. Results for Photo and No-photo images are reported in the case of NH(2, 1, 2) and NH(6, 1, 5) heuristics.

It can be observed that prediction was actually applied to the subbands obtained after RDLS-SS-DWT, which had not been reduced to DWT or to transform skipping. It was used more often when we employed more filters and predictors. The share of subbands with Mixed origin was much larger in the case of Photo images (it is the most common origin of their subbands) than for No-photo. If all transform steps were skipped for a given subband (“All Null”), then it almost always was better to apply prediction to such a subband. Skipping all steps was the most common subband origin in No-photo images. If a subband other than *LL* was obtained as in the unmodified DWT (“All None”), which was generally the least common case for both kinds of images, prediction was almost never used. In contrast, for the *LL* subband, it was almost always used, which can be explained by the fact that the characteristic of this subband was the closest to the characteristic of the original image. On average for all images and NH(6, 1, 5), prediction was used for approximately 61% of subbands. This value was roughly similar in all subbands except for *LL*, where it was used much more often (92%).

On the one hand, the above results show that DWT was effectively combined with prediction by the use of RDLS and step skipping—RDLS-SS-DWT+Pred is a hybrid transform. On the other hand, motivated by noticing how common is the use of prediction for the *LL* subband as well as in the case of “All Null” origin for all subbands and recalling effects of the heuristic’s step A (Table 2), we proposed two simple transform variants, described below. They exploit prediction, but do not use RDLS or step skipping; we investigate them in Section 3.3:

- Skipping the entire DWT and applying prediction to an untransformed image, which has not been decomposed into subbands, this variant, compared to three-level DWT with all the steps skipped and actual prediction applied to all subbands, is simpler and should obtain similar bitrates so it may be effective for many images.
- Application of the unmodified typical-level DWT with prediction applied to the *LL* subband only will almost always be better than DWT without prediction, although the difference will be small because of the small size of the *LL* subband.

### 3.2. Employing Entropy Estimation to Speed Up the Heuristic

In Table 4 we report average bitrate changes due to the hybrid transform when *H*0 is used for selecting filters in RH instead of the actual JPEG 2000 bitrate. Transform variants obtained this way, to distinguish them from the bitrate-based variants, are labeled as *H*0_RH. Compression time reported in the column labeled “Time rel.” was computed using the below $T_{\mathrm{fs}}^{H0\_\mathrm{RH}}$ instead of $T_{\mathrm{fs}}^{\mathrm{RH}}$ from Equation (12):

(14) $$T_{\mathrm{fs}}^{H0\_\mathrm{RH}}=P\left(\left(f+n\left(2f-\frac{2}{3}\right)\right)\left((p-1)c_{\mathrm{pred}}+pc_{\mathrm{est}}\right)+c_{\mathrm{enc}}\right).$$

We can see that in *H*0_RH, similarly to the bitrate-based results presented in the previous section, one iteration of step B of the heuristic was sufficient in the case of Photo images, whereas step A alone sufficed for No-photo. Compared to using all the filters and predictors, by using only two filters we negligibly worsened compression ratios and significantly improved compression speed. Also, it appears that our expectations were correct as to obtaining higher speeds and better compression ratio improvements by exploiting estimation-based selection of filters and step B of the heuristic, instead of bitrate-based selection and using only step A of the heuristic. Using only two predictors had a greater adverse effect on bitrates and smaller positive effect on speed.

### 3.3. Schemes Extending the Heuristic with Prediction Applied to Untransformed Image

In Section 3.1 we suspected that applying prediction to an unmodified image, instead of applying multiple-level DWT, may be beneficial for some images—because RDLS-SS-DWT+Pred subbands of most No-photo images were actually transformed with three-level DWT with all the steps skipped and actual prediction applied. We also suspected that applying prediction to the *LL* subband of unmodified multiple-level DWT would result in small bitrate improvements. Effects of the above variants, denoted NO-DWT+PredMED and DWT+PredMED_LL, respectively, are reported in Table 5. We use the MED predictor and did not allow choosing any other one (including NOP), as using other predictors appeared less effective in the case of untransformed images or the *LL* subband of the DWT-transformed image. Our simple entropy estimation-based method of selecting the predictor works better for high-pass filtered DWT subbands than for an untransformed image or the *LL* subband. The estimation may probably be improved by using, instead of *H*0, a conditional entropy that would better match the actual JPEG 2000 context entropy coder.

Compression ratio improvement of NO-DWT+PredMED in the case of No-photo images was little better than the results obtained using the most complex variant of the hybrid RDLS-SS-DWT+Pred (−31.49% vs. −31.19%). Obviously, DWT is not the best transform for preparing the No-photo images for entropy coding. When we used the unmodified standard JPEG 2000 coder, for about one-third of this image set (81 of 247), it was better to use JPEG 2000 with the DWT stage skipped than to apply the unmodified DWT. No-photo images contained artificially inserted sharp lines. In [16] we noticed that, due to the way 2D-DWT is built by using 1D-DWTs, sharp lines affect the unmodified DWT similarly to noisy pixels. Information on them may get propagated to the filtered subbands even in the case of the low-pass filtering of the update LS (that generally is supposed to remove the high-frequency information from the subband). The DWT efficiency can be vastly improved for these images by using RDLS, step skipping, and prediction, but using simple prediction instead of modified DWT is a better solution. NO-DWT+PredMED is not as effective for Photo images as the hybrid transform; however, we can combine the advantages of both approaches by adaptively selecting a better one for each image. Before investigating such a scheme, we noted that DWT+PredMED_LL is little better than unmodified DWT for both kinds of images without a noticeable increase in compression time.

**Figure 5:**
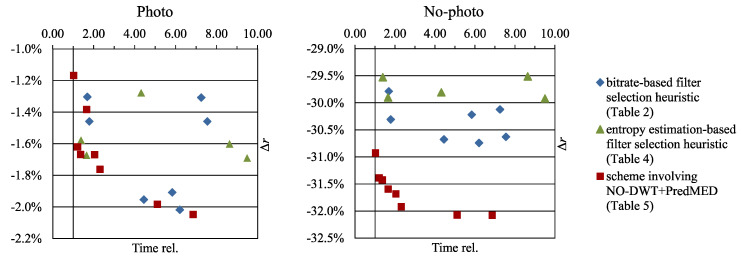
Average bitrate improvements obtained with and without NO-DWT+PredMED plotted against the cost of the improvement.

In Table 5 we also report bitrate improvements obtained by compression schemes that select, for each image, between NO-DWT+PredMED and some other earlier investigated transform variant. *H*0_sel (rows 3–5) denotes a scheme in which the selection was based on a very quick entropy estimation. We compared the entropy of the image transformed using NO-DWT+PredMED and DWT+PredMED_LL and selected NO-DWT+PredMED if it resulted in a smaller entropy. Otherwise, the other transform was selected (DWT+PredMED_LL or a variant of RDLS-SS-DWT+Pred). If NO-DWT+PredMED was selected, then the selection did not involve executing the heuristic and, thus, the compression time was shorter. Hence, for *H*0_sel we reported compression time computed by taking into account that NO-DWT+PredMED was selected for 42% of images from our test set. By min_bpp (rows 6–10), we denoted schemes in which both transform variants were performed and the one resulting in a smaller bitrate was selected. Generally, the proposed schemes, compared to RDLS-SS-DWT+Pred alone, allow further bitrate improvements that in some cases can be achieved at a reduced cost (see also Figure 5).

The effects of the two *H*0_sel schemes involving the hybrid transform (rows 4 and 5 in Table 5) are very good from a practical standpoint when we want to significantly improve compression ratios without a significant increase in compression time. By extending RDLS-SS-DWT+Pred variants obtained using *H*0_RH (recall results from Table 4) with an option of using NO-DWT+PredMED instead of RDLS-SS-DWT+Pred, we decreased the average compression time and improved compression ratios of No-photo images by over 1.5 percentage points. The faster such scheme, compared to unmodified JPEG 2000, improved bitrates of Photo and No-photo images by about 1.6% and 31.4%, respectively, at the expense of increasing the compression time on average by 21%.

The two schemes that did not use RDLS-SS-DWT+Pred and obtained lower bitrate improvements of Photo images than others (rows 3 and 6) are also interesting. These improvements were of about 1.2% to 1.4%, which, on the other hand, is better than the best improvements obtained using RDLS-SS-DWT without prediction [17]. Based on bitrate improvements and the compression time, other schemes may seem more practical but the advantage of these schemes is in their simplicity. They neither employ a heuristic nor integrate filters into transform steps. They require only minimal changes to JPEG 2000, i.e., the unconditional application of the MED predictor to the *LL* subband after performing DWT or to an untransformed image if DWT is not used. They even may be seen as a single alteration of JPEG 2000 if we consider an untransformed image to be an *LL* subband of an image transformed using the 0-level DWT.

For greater bitrate improvements than the abovementioned *H*0_sel schemes, we may select a transform based on the actual bitrate (min_bpp), use the actual bitrate instead of an estimated one for selecting filters in the heuristic (RH instead of *H*0_RH), and use a more complex heuristic (NH instead of RH). This way we attain bitrate improvements of over 2% for Photo images and over 32% for No-photo, but at the cost of compression time increased several times that may be too high for certain practical applications.

## 4. Conclusions and Further Work

It is believed that the use of prediction that is not supported by additional knowledge does not significantly improve the lossless compression ratios of DWT subbands. However, because the characteristic of RDLS-SS-DWT subbands is different, we proposed a hybrid transform for lossless image compression, named RDLS-SS-DWT+Pred, that was obtained by applying prediction to RDLS-SS-DWT subbands. The transform was constructed in an image-adaptive way; the predictor function for each subband was selected based on memoryless entropy of the subband and we tested two heuristics (NH and RH) for the selection of RDLS filters. Experiments were performed for a large and diverse test set consisting of 499 Photo and 247 No-photo (screen content) images.

We found that RDLS with step skipping allows DWT to be effectively combined with prediction, which results in significant improvements of lossless JPEG 2000 compression ratios. Both for Photo and No-Photo images, the average improvement due to RDLS-SS-DWT+Pred was nearly twice as high as that obtained using RDLS-SS-DWT without prediction. The hybrid transform was especially effective for No-photo images. The cost of the compression ratio improvement can be significantly reduced by using RH with a single iteration of the heuristic’s step B, by allowing only special filters (None and Null) and by exploiting *H*0 as an estimator of JPEG 2000 entropy coding effects for the selection of filters for RDLS.

Because for certain images (especially for No-photo) it may be better to use NO-DWT+PredMED, we proposed the below practical compression schemes.

- The scheme using NO-DWT+PredMED and RDLS-SS-DWT+Pred, compared to the unmodified JPEG 2000, improves the compression ratios of Photo and No-photo images by about 1.6% and 31.4%, respectively, at the expense of a small increase in the compression time (by 21%). Larger bitrate improvements, exceeding 2% and 32%, respectively, are possible at the expense of increasing the compression time several times.
- The scheme employing DWT+PredMED_LL instead of RDLS-SS-DWT+Pred is also interesting despite improving compression ratios to a smaller extent. It can be applied by introducing only minimal and simple modifications to JPEG 2000 and allows for improving the bitrates by 1.2% and 30.9%, respectively, at a negligible cost of 2% compression time increase.

It would be interesting to apply the proposed hybridization method to compression algorithms based on different transforms. For example, it would be technically possible to apply RDLS and prediction to the reversible DCT employed by lossless JPEG-XR. Yet, obtaining for JPEG-XR similar compression ratio improvements as for JPEG 2000 may not be easy. The characteristic of the DCT-transformed image data seems much less feasible for prediction and DCT is in JPEG-XR used for small fragments of the image. Thus, RDLS and prediction would cause greater overhead due to transmitting the filters and predictors to the decoder.

In ongoing research, we are investigating an application of RDLS-SS-DWT+Pred to the 3-dimensional DWT employed by JP3D in lossless compression of volumetric medical images as well as RDLS filter selection using the DPC model in the case of RDLS-modified color space transforms [38]. The effectiveness of the memoryless entropy as an estimator of coding effects that we used for adaptive selection of filters and predictors may be improved by using, instead of *H*0, a conditional entropy better matching the actual JPEG 2000 context entropy coder. We also plan to investigate sophisticated denoising filters for RDLS, to combine RDLS-SS-DWT+Pred with RDLS-modified color space transforms in lossless compression of color images, and to apply our methods in compression of raw color filter array image data acquired by digital cameras.

## Figures and Tables

**Table 2:** Effects of integration of prediction with DWT and RDLS-SS-DWT.

	Transform Variant	Time	Images	
		Rel.	Photo	No-Photo
1	*r*_DWT_	1.00	3.9975	2.9162
2	*∆r*_DWT+Pred(5)_	1.07	−0.18%	−1.33%
3	*∆r*_RDLS-SS-DWT, NH(6,2,1)_	55.85	−1.08%	−17.59%
4	*∆r*_RDLS-SS-DWT+Pred, NH(6,2,5)_	59.32	−2.08%	−31.19%
5	*∆r*_RDLS-SS-DWT+Pred, NH(6,1,5)_	33.43	−2.05%	−31.16%
6	*∆r*_RDLS-SS-DWT+Pred, NH(6,0,5)_	7.55	−1.46%	−30.63%
7	*∆r*_RDLS-SS-DWT+Pred, NH(6,1,2)_	32.08	−1.94%	−30.66%
8	*∆r*_SS-DWT+Pred, NH(2,1,5)_	6.20	−2.02%	−30.74%
9	*∆r*_SS-DWT+Pred, NH(2,1,2)_	5.84	−1.91%	−30.22%
10	*∆r*_SS-DWT+Pred, RH(2,1,5)_	4.45	−1.95%	−30.68%
11	*∆r*_RDLS-SS-DWT+Pred, NH(6,0,2)_	7.25	−1.31%	−30.12%
12	*∆r*_SS-DWT+Pred, NH(2,0,5)_	1.79	−1.46%	−30.31%
13	*∆r*_SS-DWT+Pred, NH(2,0,2)_	1.69	−1.30%	−29.79%

Note: The variant’s compression time relative to the time of unmodified JPEG 2000 (Time rel.) is expressed without unit of measurement; the compression ratio of unmodified JPEG 2000 (*r*_DWT_) is expressed in bpp, whereas the bitrate changes obtained due to introducing transform variants (*∆r*_variant_) are expressed in percentages of *r*_DWT_; besides variants introduced herein, the effects of applying non-hybrid RDLS-SS-DWT are reported in row 3.

**Table 3:** Execution times of elements of JPEG 2000 and the heuristic.

Description	Time (ms per 10^6^ Pixels)	Percentage of *T_J_*
Unmodified JPEG 2000 compression (*T_J_*)	258.6	100.0%
3-level DWT transform	19.7	7.6%
Entropy coding	167.0	64.6%
Remaining JPEG 2000 operations	72.0	27.8%
Entropy estimation	1.3	0.5%
Prediction (MED predictor)	3.0	1.1%
Denosing	62.7	24.3%

**Table 4:** Effects of employing entropy estimation in RH.

	Transform Variant	Time	Images	
		Rel.	Photo	No-Photo
1	*∆r*_RDLS-SS-DWT+Pred,_ *_H_*_0_RH(6,2,5)_	14.67	−1.71%	−29.92%
2	*∆r*_RDLS-SS-DWT+Pred,_ *_H_*_0_RH(6,1,5)_	9.50	−1.69%	−29.92%
3	*∆r*_RDLS-SS-DWT+Pred,_ *_H_*_0_RH(6,0,5)_	4.32	−1.28%	−29.81%
4	*∆r*_RDLS-SS-DWT+Pred,_ *_H_*_0_RH(6,1,2)_	8.64	−1.60%	−29.51%
5	*∆r*_SS-DWT+Pred,_ *_H_*_0_RH(2,1,5)_	1.65	−1.67%	−29.90%
6	*∆r*_SS-DWT+Pred,_ *_H_*_0_RH(2,1,2)_	1.39	−1.58%	−29.53%

Note: Symbols and units of measurement—see Table 2 note.

**Table 5:** Exploiting application of prediction to an unmodified image or to the *LL* subband of the DWT-transformed image.

	Transform Variant/Scheme	Time	Images	
		Rel.	Photo	No-Photo
1	*∆r*_NO-DWT+PredMED_	0.94	−0.19%	−31.49%
2	*∆r*_DWT+PredMED_LL_	1.00	−0.24%	−0.82%
3	*∆r_H_*_0_sel(NO-DWT+PredMED; DWT+PredMED_LL)_	1.02	−1.17%	−30.93%
4	*∆r_H_*_0_sel(NO-DWT+PredMED; SS-DWT+Pred,_ *_H_*_0_RH(2,1,2))_	1.21	−1.62%	−31.39%
5	*∆r_H_*_0_sel(NO-DWT+PredMED; SS-DWT+Pred,_ *_H_*_0_RH(2,1,5))_	1.36	−1.67%	−31.43%
6	*∆r*_min_bpp(NO-DWT+PredMED; DWT+PredMED_LL)_	1.66	−1.38%	−31.59%
7	*∆r*_min_bpp(NO-DWT+PredMED; SS-DWT+Pred,_ *_H_*_0_RH(2,1,2))_	2.04	−1.67%	−31.68%
8	*∆r*_min_bpp(NO-DWT+PredMED; SS-DWT+Pred,_ *_H_*_0_RH(2,1,5))_	2.31	−1.76%	−31.92%
9	*∆r*_min_bpp(NO-DWT+PredMED; SS-DWT+Pred, RH(2,1,5))_	5.11	−1.98%	−32.07%
10	*∆r*_min_bpp(NO-DWT+PredMED; SS-DWT+Pred, NH(2,1,5))_	6.86	−2.05%	−32.08%

Note: Symbols and units of measurement—see Table 2 note.

## Supplementary Materials

The following is available online at https://www.mdpi.com/1099-4300/22/7/751/s1, Supplementary File S1: RDLS-SS-DWT+Pred Implementation, Version 1.0.

## Nomenclature

⌊v⌋	the greatest integer less than or equal to *v*
*∆* *r*_variant_	average bitrate change due to applying specific transform variant
*c*_den_	cost of denoising of a sample
*c*_enc_	cost of entropy coding of a single prediction error
*c*_est_	cost of estimating bitrate per single sample
*c*_LS_	cost of a single LS
*c*_pred_	cost of predicting a sample
*f*	number of filters
*l*	transform level
*M*	number of samples in the subband
MaxPE	the greatest prediction error obtained
MinPE	the smallest prediction error obtained
N	upper neighbor of the pixel being predicted
*n*	number of iterations of the step B of the heuristic
NW	upper-left neighbor of the pixel being predicted
*P*	size of the image (number of pixels)
*p*	number of predictors
*p_i_*	probability of occurrence of the prediction error value *i* in the subband
*q*	signal length
*r*_DWT_	average bitrate of unmodified JPEG 2000
*S*	discrete signal
$s_{i}$	*i*-th signal sample
$s_{i}^{d}$	denoised sample $s_{i}$
$T_{\mathrm{as}}^{\mathrm{NH}}$	cost of applying RDLSs while computing all subbands by NH
$T_{\mathrm{as}}^{\mathrm{RH}}$	cost of applying RDLSs while computing all subbands by RH
$T_{\mathrm{fs}}^{H0\text{\_}\mathrm{RH}}$	cost of applying prediction and entropy coding of final subbands by entropy estimation-based variant of RH
$T_{\mathrm{fs}}^{\mathrm{NH}}$	cost of applying prediction and entropy coding of final subbands by NH
$T_{\mathrm{fs}}^{\mathrm{RH}}$	cost of applying prediction and entropy coding of final subbands by RH
*T_J_*	time of unmodified JPEG 2000 compression
W	left-hand neighbor of the pixel being predicted
